# Ask A Biologist: Bringing Science to the Public

**DOI:** 10.1371/journal.pbio.1000458

**Published:** 2010-10-12

**Authors:** Charles Kazilek

**Affiliations:** School of Life Sciences, Arizona State University, Tempe, Arizona, United States of America

## Abstract

Puzzling questions about science spur millions to the virtual doorstep of Ask A Biologist, an educational website that proves knowledge-building is a growth industry.

In the early days of the Internet, before Google-powered searches retrieved information on even the most arcane subjects with a single keystroke, inquiring minds sought the advice of experts directly. A critical online intermediary between seekers and experts was the “Ask a (fill in the blank)” website. “Ask a” sites put users with burning questions to solve in direct contact with experts on everything from hornets and cures for rug burn to stellar explosions and baseball trivia. Recognition of the “ask a question” tool's potential in the rapidly evolving electronic frontier led to Web offerings such as The Mad Scientist Network, Howard Hughes Medical Institute's Ask a Scientist, and *Scientific American's* Ask the Experts.

Ask A Biologist (askabiologist.asu.edu) was an early adopter of this approach. Although conceptually similar to the expert phone lines established by libraries and universities across the country prior to the creation of the Web, “Ask a” websites can provide 24-hour service without requiring 24-hour staffing while reaching a global audience. Users need just Internet access and, in some cases, an email address, to enjoy streamlined information retrieval, often getting answers within days or even hours. The “Ask a” sites changed the relationship the public could have with experts, and with cutting-edge science.

“Ask a” websites also helped the scientific community communicate with students, teachers, and life-long learners on their own time. An expert could take the extra time and care to craft the most suitable reply, for example, when challenged to write a grade-appropriate answer to an elementary school student, or to locate additional resources for a teacher to use in the classroom.

Since 1997, Ask A Biologist has grown from a single page on Arizona State University's School of Life Sciences website to more than 2,500 pages of content. More than 150 scientists and experts support the “Ask a” section, which has now offered insight to more than 25,000 perplexed or curious children and adults. The School of Life Sciences is the home for a large group of biology experts who can provide insights on a wide range of topics. Questions are routed to appropriate faculty and graduate student experts through a common email address which protects any single person from being inundated with questions. It also provides a level of review, and an opportunity to revise answers to ensure they are grade appropriate.

In addition to the core question and answer (Q & A) feature, a strong conduit between the public and the working scientist, Ask A Biologist has grown to involve scientists in content creation. The site has also evolved to include multiple media formats. Audio interviews with scientists, video, teachers' tools, photo galleries, and games have been developed to accommodate different types of learners and meet the expectations of nearly one million visitors, yearly (Figure 1).

**Figure 1:**
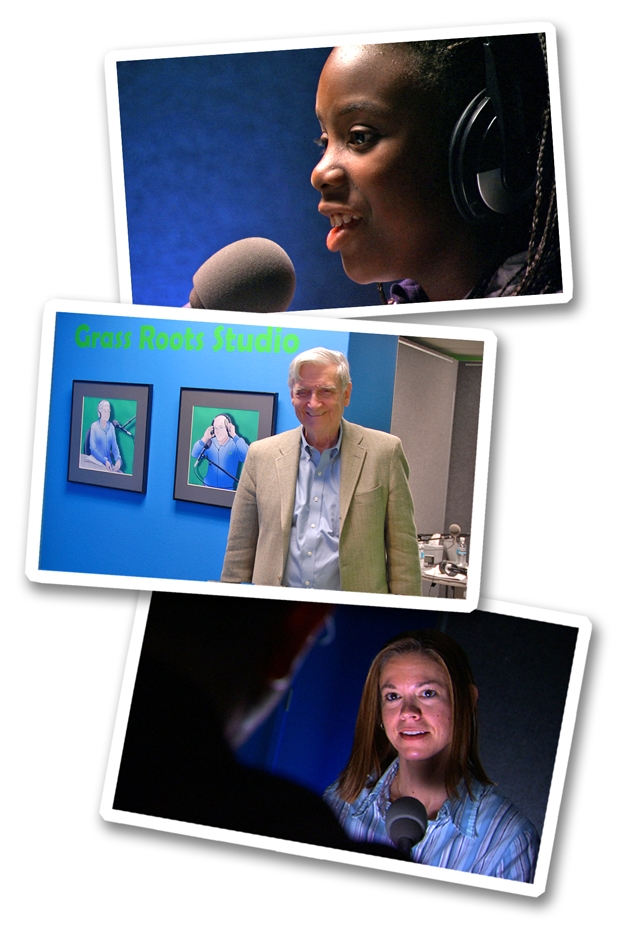
Talking Science in the Grass Roots Studio—Ask A Biologist podcast co-host and guests: (from top to bottom) future scientist Taylor Cheatham (Wickedly Cool Plants interview), Edward O. Wilson (Science Rock Star interview), and rising star microbiologist Shelly Haydel (Mud Science - Healing with Clays interview). Photos: Actual Films and CJ Kazilek.

Like other “Ask a” sites, the Ask A Biologist website has evolved over the years to compete with the continual expansion of the Web's content and search tools. Today, it's typically not the lack of information that brings people to Ask A Biologist, but instead the huge volume of information to be found on a single site. A Web search can offer multiple, differing or contradictory answers. This expansion of possibilities invites more questions, especially about which answer is, or answers are, correct? These new challenges have changed both the role of the “Ask a” site and the types of questions received.

Equally important are the questions regarding not a topic in science, but the people and careers in science. More than one quarter of the questions that Ask A Biologist receives are from students who want to learn more about careers in biology. They want to know what it is like to walk in the shoes of someone who studies pika in China or shapes policy around fishing in the Great Lakes or manipulates algae to solve problems in bioenergy. Students want to know how different biologists shaped their careers, what classes they took, who influenced their lives, and what opportunities opened doors to their dreams. These career path questions are as important to us as to the student who wants to find the solution to the emerging challenges in disease, climate change, and our environment.

Maintaining a connection with the public is critical to any website, but especially an educational website. The Q & A submission remains a powerful tool for deciding which activities or content to build on, typically based on popularity. Our quiz feature emerged in response to requests from our student users, for example. Our coloring pages for early and non-readers were created at the request of parents and grandparents looking for activities for young charges.

The Q & A tool also alerted us to a growing problem with student language skills. While students submitted startlingly good science questions, their language and grammar skills were in decline. Though this program focuses on science, communication skills are of equal concern. The launch of the Mysterious World of Dr. Biology (Figure 2) online comic book adventure activity, which uses the popular comic genre as a vehicle for storytelling, was developed to meet this growing challenge. The activity provides teachers and students a collection of clipart that can be used in paper or electronic form to create their own adventures.

**Figure 2:**
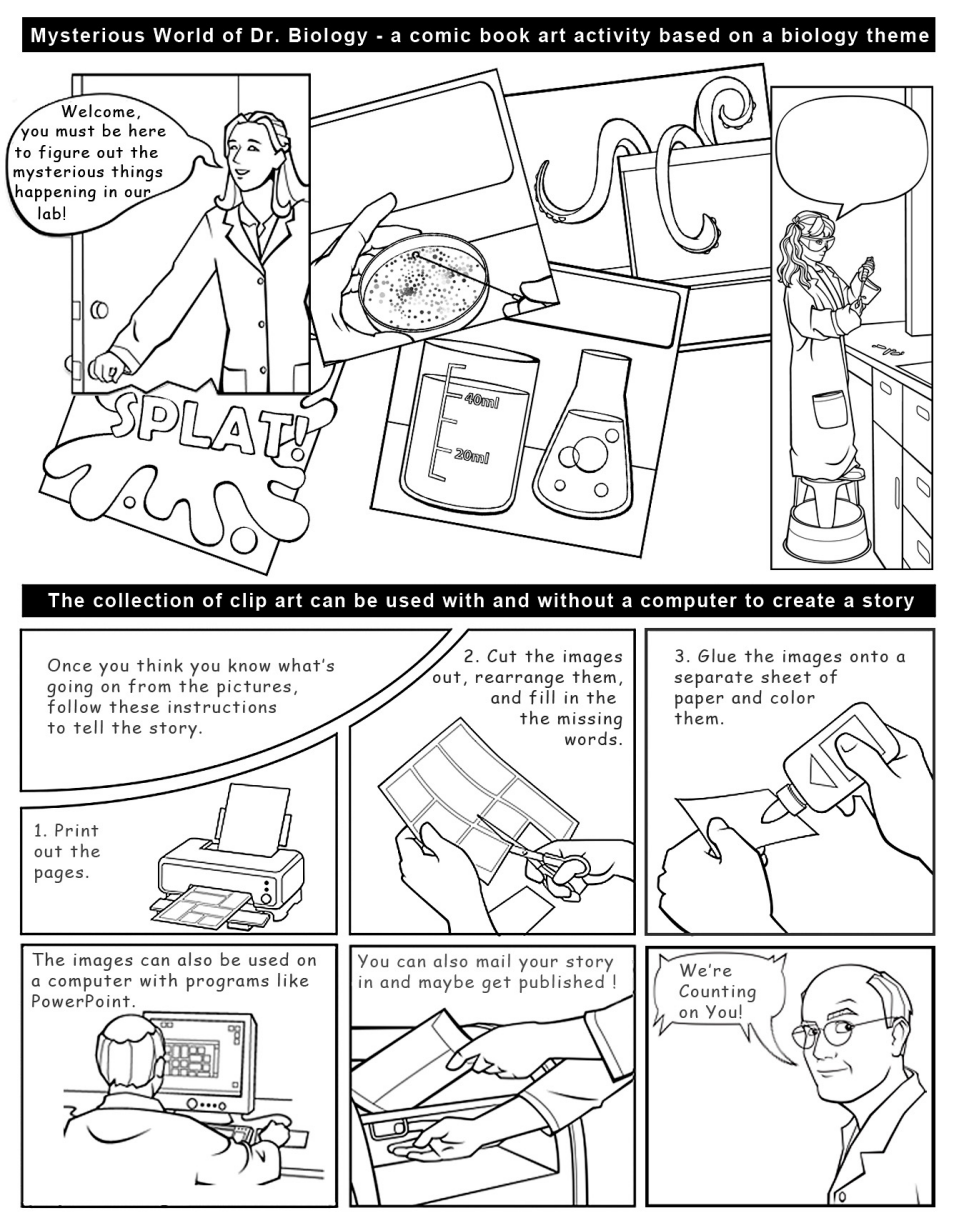
Mysterious World of Dr. Biology—Students can create their own adventure using the biology-focused clip art collection. The activity encourages writing and story development. Illustrations: Sabine Deviche.

The hunger for multimedia has also led to the expansion of Ask A Biologist. The site now has several new video companion channels including, YouTube, TeacherTube, and Vimeo. To bring the voice of the scientist to life there is an Ask A Biologist iTunes U audio channel with over 50 episodes of interviews and transcripts. Guests range from Pulitzer Prize-winning authors to graduate students at the beginning of their science careers. An equally exciting feature of the podcast is a contest that brings K-12 students, their teachers, and parents to the ASU campus for a day of discovery and the opportunity to co-host the program. Winners of the contest not only have a day of science, they also become part of iTunes, with their name and episode rubbing elbows with their favorite music or movie stars.

Flexible approaches to learning also allow Ask A Biologist to provide experiments and activities that can be used with and without a computer – expanding the reach of the site beyond the Web. One such activity is “Dr. Biology's Virtual Pocket Seed Experiment” (Figure 3). This seed germination experiment and its “fun, flexible datasets” can be presented in a virtual format in a classroom. The virtual experience includes multiple experimental treatments. Each treatment covers 10 days and includes time-lapse animation. The experiment can also be downloaded in PDF format, printed, and used as a hands-on experiment. Teachers are able to use the tools in concert, using the virtual version with its animations to capture the imagination of the students, followed by the tactile version that students control. This type of activity has proven to be popular, with more than 500 downloads of the PDF experiment each month including a companion version in Spanish.

**Figure 3:**
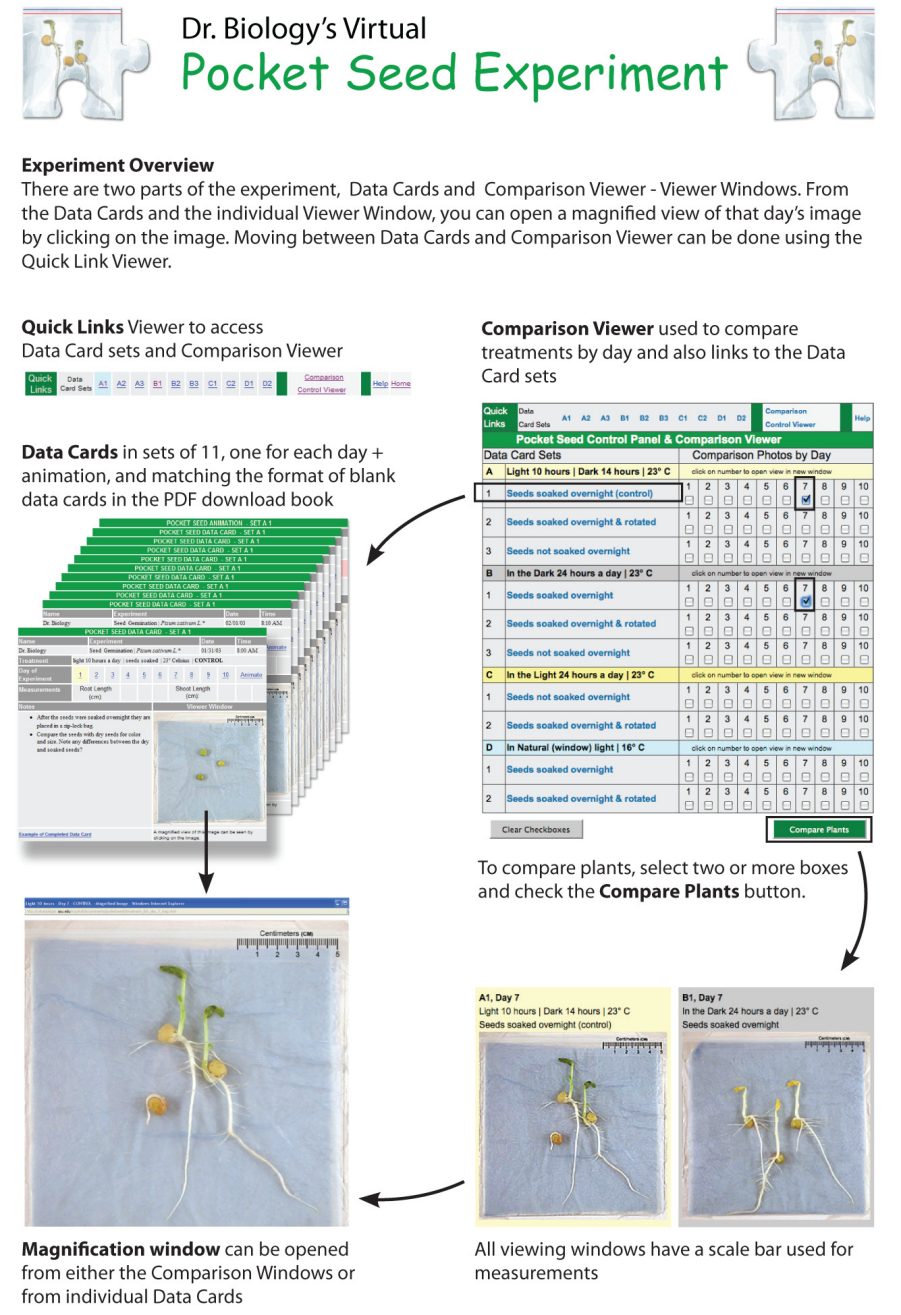
Virtual Pocket Seed Experiment—An online activity that includes a large collection of experimental data including images, animations, classroom activity packet, and companion Excel file for entering and graphing results (available in English, Spanish and French).

Recognizing the need for new avenues of learning, as well as new means of collaborative exchange has enhanced Ask A Biologist's evolution as a learning tool. A major transformation in look and features was made possible thanks to a recent grant from the National Science Foundation (NSF) and the National STEM Distributed Learning (NSDL) program. Support for the site now includes new volunteers, including illustrators, science writers and language translators, in addition to scientists. Partnerships with other groups have also expanded possibilities. For example, the Ask A Biologist program is working with the Arizona Science Center to build and host a Web version of their Body Depot gallery program. Part of the Framing New Pathways to Medical Discovery grant funded by the National Center for Research Resources (NCRR), the project introduces students aged 8–14 years and their teachers to three biomedical research areas inspired by NIH's Roadmap for Medical Research: Biological Pathways, Bioinformatics, and Nanomedicine using the metaphor of a hardware store to explain how the body maintains and repairs itself.

While the Ask A Biologist program has undergone substantial changes during its 13 years, it has stayed true to the original focus, building that link between the public and working scientists. It provides a window into the possibilities that science offers, fosters inspiration and, perhaps, the means for every user, young or old, male or female, student or teacher, to imagine and potentially reach their own dreams. Such inspiration, created in concert with the public, means Ask A Biologist, and “Ask a” sites like it, may be here to stay, growing and adapting to the needs of students, teachers, and life-long learners, long into the future.

